# Effects of non-invasive brain stimulation in children and young people with psychiatric disorders: a protocol for a systematic review

**DOI:** 10.1186/s13643-021-01627-3

**Published:** 2021-03-11

**Authors:** Yael D. Lewis, Lucy Gallop, Iain C. Campbell, Ulrike Schmidt

**Affiliations:** 1 Section of Eating Disorders, Department of Psychological Medicine, Institute of Psychiatry, Psychology and Neuroscience, King’s College London, PO59, London, SE5 8AF UK; 2 Hadarim Eating Disorder Unit, Shalvata Mental Health Centre, Hod Hasharon, Israel; 3 Sackler Faculty of Medicine, Tel-Aviv University, Tel-Aviv, Israel; 4 South London and Maudsley NHS Foundation Trust, London, UK

**Keywords:** Neuromodulation, Non-invasive brain stimulation (NIBS), Repetitive transcranial magnetic stimulation (rTMS), Transcranial direct current stimulation (tDCS), Children and adolescents, Young people

## Abstract

**Background:**

Most psychiatric disorders have their onset in childhood or adolescence, and if not fully treated have the potential for causing life-long psycho-social and physical sequelae. Effective psychotherapeutic and medication treatments exist, but a significant proportion of children and young people do not make a full recovery. Thus, novel, safe, brain-based alternatives or adjuncts to conventional treatments are needed. Repetitive transcranial magnetic stimulation (rTMS) and transcranial direct current stimulation (tDCS) are non-invasive brain stimulation (NIBS) techniques which have shown clinical benefits in adult psychiatric conditions. However, in children and young people their efficacy is not well established. The objective of this study will be to systematically evaluate the evidence on clinical effects of NIBS in children and young people with psychiatric disorders, assessing disorder-specific symptoms, mood and neurocognitive functions.

**Methods:**

We designed and registered a study protocol for a systematic review. We will include randomised and non-randomised controlled trials and observational studies (e.g. cohort, case-control, case series) assessing the effects of NIBS in children and young people (aged ≤ 24 years old) for psychiatric disorders. The primary outcome will be reduction of disorder-specific symptoms. Secondary outcomes will include effects on mood and cognition. A comprehensive search from database inception onwards will be conducted in MEDLINE, EMBASE and PsycINFO. Grey literature will be identified through searching multiple clinical trial registries. Two reviewers will independently screen all citations, full-text articles and abstract data. The methodological quality of the studies will be appraised using appropriate tools. We will provide a narrative synthesis of the evidence and according to heterogeneity will conduct an appropriate meta-analysis. Additional analyses will be conducted to explore the potential sources of heterogeneity.

**Discussion:**

This systematic review will provide a broad and comprehensive evaluation of the evidence on clinical effects of NIBS in children and young people with psychiatric disorders. Our findings will be reported according to the PRISMA guidelines and will be of interest to multiple audiences (including patients, researchers, healthcare professionals and policy-makers). Results will be published in a peer-reviewed journal.

**Systematic review registration:**

PROSPERO CRD42019158957

**Supplementary Information:**

The online version contains supplementary material available at 10.1186/s13643-021-01627-3.

## Background

Mental health disorders affect 10–20% of the child and adolescent population [1]. In a recent population-based survey, one in eight (12.8%) 5- to 19-year olds in England was found to have at least one mental disorder [2]. In a large multi-national self-report survey initiated by the World Health Organisation (WHO), a third of first-year university students screened positive for one of the major anxiety, mood or substance disorders [3]. Importantly, 50% of all adult mental health disorders emerge before 14 years of age and 75% by 25 years [4]. Thus, the disease burden of such disorders, starting from childhood into emerging adulthood, is considerable in earlier as well as later decades of life, with protracted adverse outcomes in educational attainment, employment, physical health and social functioning [1, 5, 6].

The appearance of psychiatric disorders during childhood/adolescence coincides with significant neurodevelopmental processes identified by longitudinal neuroimaging studies [7]. These include a general decrease in grey matter volume from a childhood peak an increase in white matter volume, alongside a reported imbalance between the dominant limbic and reward systems which develop first, and the executive prefrontal system which matures later [8–10]. These processes continue until the early-mid 20s [11, 12], in accordance with the more extensive and psychosocially based definitions of adolescence and emerging adulthood [13, 14]. Neurobiological dynamics, and the limbic-executive maturation gap in particular, have been proposed to contribute to the vulnerability to psychopathology [15], although further study is warranted [16].

The importance of early detection and intervention is supported by the evident neuroplasticity of the adolescent brain (which provides a window for impacting development [17]) and by clinical studies showing that early intervention improves outcome, e.g. in psychosis [18] and in eating disorders [19, 20]. Treatment guidelines for psychiatric disorders in children and young people focus on psycho-social interventions (including psychotherapies) and psychopharmacological treatments [21]. However, outcomes are variable, and only partially meet existing needs. While psychotherapy for children and young people has shown significant benefits in research trials [22], effects are considerably diminished in routine clinical settings [23]. Pharmacotherapy for children and young people is not well-established for many disorders [24], and adult-approved pharmacological treatments are often given “off-label” with little supportive evidence [25]. There are also significant unresolved concerns over the safety of pharmacotherapy in children and young people [26]. Given the limitations of current treatment modalities for psychiatric disorders in children and young people, researchers are highlighting the need for novel biotherapies that can be used safely, as additions or alternatives to established, conventional interventions in youth mental health [27].

Transcranial magnetic stimulation (TMS) is a non-invasive procedure used to modulate cortical excitability in target brain regions: it is generally considered to be safe [28]. In TMS, an electromagnetic coil is used to generate a magnetic field that passes through the skull and induces a current in the underlying neural tissue, which depolarises neurones [29]. Single- and paired-pulse TMS can temporarily affect motor, sensory or cognitive behaviour [30] and repetitive TMS (rTMS) can induce changes in neural activity that outlast the rTMS train [31] with more durable changes reported when rTMS is given daily for 1–6 weeks [32].

A widely accepted mechanism for rTMS- and theta burst stimulation (TBS)-induced changes in synaptic efficacy is the long-term potentiation/depression (LTP/LTD) of excitatory synaptic transmission [33]. Indeed, findings have shown that rTMS and TBS in adults can be effective in improving symptoms in neuropsychiatric disorders associated with cerebral hyper- or hypo-excitability, including schizophrenia [34–36], eating disorders [37] and obsessive-compulsive disorder [38, 39]. Evidence in major depressive disorder (MDD) is the most established [40, 41] and has been incorporated into clinical guidelines [42, 43]. Pooled analyses of rTMS trials identified young age as a predictor for higher efficacy of rTMS in depression [44] and for auditory hallucinations in schizophrenia [45].

In children and young people, preliminary results indicate benefits of rTMS in treatment-resistant depression [46–48], attention deficit hyperactivity disorder (ADHD) and autism spectrum disorder (ASD) [49, 50]. For example, Hett et al. [48] found that all 14 studies included in the review reported that rTMS had some effect at reducing symptoms of depression in adolescents. Additionally, Masuda et al. [49], suggested that rTMS may ameliorate ASD symptoms (e.g. lethargy) that conventional treatments have failed to address. However, the absence of sham-controlled randomised trials and lack of rigorous treatment protocols is consistently noted in systematic reviews. Systematic reviews suggest that the safety profile of rTMS is comparable to that found in adults, with most adverse events being mild and overall uncommon [51, 52].

Transcranial direct current stimulation (tDCS) is another well-established non-invasive neuromodulation technique. It involves application of a constant weak direct current via electrodes placed on the scalp [53]. The current applied is subthreshold and unlike rTMS, it cannot induce neuronal firing, but rather modulates existing neuronal activity by changing excitability and discharge, i.e. it is affected by brain activity at the time of the stimulation [54]. Generally, cathodal stimulation results in decreased cortical excitability whereas anodal stimulation increases it [55]. Due to the relative ease of use and its safety, it has been studied extensively as a means of cognitive enhancement and behavioural modulation [53], as well for clinical therapeutic effects across psychiatric/neurological disorders [56].

Previous reviews have looked at the application of tDCS across psychiatric disorders in adults [57–59]: beneficial effects have been demonstrated in depression and schizophrenia in particular, as well as the absence of serious adverse events. An evidence-based analysis of clinical trials until 2016 by the European Chapter of the International Federation of Clinical Neurophysiology, found ‘probable efficacy’ in fibromyalgia, non-resistant depression and craving/addiction [56].

With regard to paediatric populations, a systematic review by Buchanan et al. [60], found that overall the safety evidence appears to be strong and consistent for 10- to 20-min tDCS sessions ranging from 0.5 to 2 mA in ages 5–18. These findings are in keeping with previous reviews, including Muszkat et al. [61] who identified six studies of tDCS in children and adolescents with psychiatric disorders. The authors concluded that the technique may be well tolerated and safe but that efficacy could not be established. A later more comprehensive review by Palm et al. [62] included studies on neurological and psychiatric disorders and found positive clinical effects in ADHD and ASD. They also emphasised the dearth of data for tDCS treatment of other psychiatric disorders in children and adolescents, particularly for depression and schizophrenia. More recent narrative reviews [63, 64] have reported similar conclusions, emphasising the rapid expansion of research, with over a dozen registered trials in ClinicalTrials.gov and several completed unpublished trial, but without giving further details on these. Thus, emerging study data are anticipated.

In summary, systematic reviews are available that have examined the safety profile of rTMS and tDCS in children and adolescents [51, 52]. Other reviews have summarised clinical efficacy in psychiatric conditions but have not followed rigorous methodology for systematic reviews [64, 65], require updating [61] or have focused exclusively on a specific condition [47, 49]. Furthermore, available reviews have not included unpublished data. As this is a rapidly developing field, relevant to clinicians and researchers, a broad, up-to-date systematic review encompassing published and unpublished data is required. Our primary aim is to examine the disorder-specific effects of rTMS and tDCS as therapeutic interventions in the treatment of different psychiatric disorders in children and young people. Our secondary aim is to assess broader effects of these interventions on mood and cognitive functioning in children and young people with mental health problems. Lastly, we will review application methods for both techniques such as coil modalities and stimulation parameters, in order to synthesise data on available efficacious and safe rTMS and tDCS protocols.

### Objectives

This study will systematically review available data on past, ongoing and upcoming studies using rTMS or tDCS as a therapeutic intervention in children and young people (age ≤ 24 years), with psychiatric disorders. The age range is extended to 24 years as continued neurodevelopment occurs until the mid-20s, particularly in fronto-limbic systems [66, 67]. This review will address the following questions:

1. What are the effects of rTMS and tDCS on disorder-specific symptoms in children and young people with different psychiatric disorders?
2. In children and young people with disorders other than mood disorders, what are the effects of rTMS and tDCS on mood?
3. What are the effects of rTMS and tDCS on neurocognition in this population?
4. What stimulation parameters have been used in rTMS and tDCS administration and how have these affected results?
5. What populations and stimulation parameters methods are being used in ongoing studies?

## Methods

The present study protocol is being reported in accordance with the reporting guidance provided in the Preferred Reporting Items for Systematic Reviews and Meta-Analyses Protocols (PRISMA-P) statement [68] (see PRISMA-P checklist in Additional file 1). This protocol has been registered within the International Prospective Register of Systematic Reviews (PROSPERO) database (registration ID CRD42019158957). Any amendments made to this protocol when conducting the study will be outlined in PROSPERO and reported in the final manuscript. The proposed systematic review and meta-analysis will be reported in accordance with the reporting guidance provided in the PRISMA statement [69].

### Eligibility criteria

Studies will be included according to the following criteria: participants, interventions and comparators, outcome(s) of interest and study design.

#### Participants

We will include studies involving children and young people (aged ≤ 24 years old) with all major psychiatric disorders typically affecting this age group. Eligible psychiatric disorders (ICD-10 code) will be autism spectrum disorder (F84), attention deficit hyperactivity disorder (F90), conduct disorders (F91), impulse control disorders (F63), schizophrenia (F20), bipolar disorder (F31), depression (F32 and F33), anxiety disorders (F40 and F41), obsessive-compulsive disorder (F42), tic disorders (F95), posttraumatic stress disorder (F43), substance abuse disorder (F10–F19), somatoform disorders (F45), eating disorders (F50) and personality disorders (F60). We will exclude studies in non-clinical populations.

#### Interventions

Multiple session (sessions ≥ 2) studies using tDCS and rTMS for a clinical purpose will be included. For rTMS studies, we will include all variants of rTMS administered, including low-frequency rTMS (LF-rTMS), high-frequency rTMS (HF-rTMS), intermittent theta burst stimulation (iTBS), continuous theta burst stimulation (cTBS), paired associative stimulation (PAS), repetitive paired-pulse stimulation (PPS) or quadripulse stimulation (QPS).

#### Comparators

Sham stimulation or treatment as usual. For some reports there may be no comparison (open-label trials, case reports, case series).

#### Outcomes of interest

The primary outcome will be disorder-specific clinical outcomes, as measured by a standardised assessment tool pre-and post-intervention, e.g., the Positive and Negative Syndrome Scale (PANNS) for schizophrenia or described narratively. Secondary outcomes will be (1) change in mood as measured by a standardised assessment tool pre- and post-intervention, e.g., Hamilton Depression Rating Scale (HDRS); (2) change in neurocognitive functioning, e.g., Iowa Gambling Task (IGT); and (3) reported adverse outcomes including side effects.

#### Study design

We will consider randomised controlled trials, non-randomised control trials, open-label trials, crossover trials, cohort, case-control, case series and case reports.

Only studies published in English will be included. No limitations will be imposed on publication status (unpublished studies will be eligible for inclusion) or study conduct period.

### Information sources and search strategy

The primary source of literature will be a structured search of electronic databases (from their inception onwards): MEDLINE, EMBASE and PsycINFO. The secondary source of potentially relevant material will be a search of clinical trial registries including the WHO International Clinical Trials Registry Platform (ICTRP) registry, ClinicalTrials.gov, the National Institute of Health (NIH) registry, the European Union Clinical Trials Register and the International Standard Randomised Controlled Trials Number (ISRCTN) registry. We will perform hand-searching of the reference lists of included studies and relevant reviews. The literature searches will be designed and conducted by the review team with the assistance of an experienced health information specialist. Our main literature search will be peer-reviewed by a senior health information specialist using the Peer Review of Electronic Search Strategies (PRESS) checklist [70]. The search will include a broad range of terms and keywords related to children and young people, psychiatric disorders and non-invasive brain stimulation (NIBS). A draft search strategy for MEDLINE is provided in Additional file 2.

### Study selection

All articles yielded by the searches will be uploaded on to Rayyan QCRI web application which will be used for the selection process [71]. Two authors will independently screen all titles and abstracts for inclusion criteria. If eligibility cannot be ascertained from the title or abstract, the full text will be examined. Any disagreement regarding the included articles will be resolved through discussion, and if necessary, a third reviewer from the team will be consulted. After the initial screening, duplicates will be removed and the remaining articles will be reviewed in full-text form for inclusion and data extraction. Documentation regarding the source of article (database/trial registry/manual reference check), its inclusion or exclusion and the reasoning will be recorded and presented in the PRISMA flowchart.

### Data extraction

Two authors will extract data independently and in duplicate from the included articles, using a purpose-developed form adapted from the Cochrane data collection form for intervention reviews [72] (see Additional file 3). This form was piloted on several papers obtained from a preliminary search. The extracted data will be recorded in spreadsheets using Excel software. Extracted data will include the following items:

- *Study characteristics*. Title, reference citation, publication type, language of publication, study design and a priori sample size calculation
- *Participants/population*. Number of participants, age, gender, ethnicity, inclusion/exclusion criteria, main disorder, treatment setting, severity of illness, co-morbidities and subgroup division (e.g., according to age or disorder subtype like attention deficit disorder with and without hyperactivity)
- *Intervention*. Concomitant treatments participants received during intervention, e.g. medication or psychotherapy, brain-manipulation during stimulation, e.g. cognitive or other task which may have been completed during stimulation
- *rTMS*. Type of rTMS used, coil type, site of stimulation, neuro-navigation use, stimulation intensity, session duration and frequency, total number of sessions and compliance with rTMS regimen
- *tDCS*. Stimulation electrode location, current intensity and duration, session number and frequency and total number of sessions
- *Comparators*. Sham, treatment as usual, waitlist and no comparison
- *Outcomes*. Dropout, main disorder-specific assessment tool and results, mood assessment tool and outcome, cognitive assessment tool and outcomes and adverse effects reported

### Risk of bias in individual studies

Risk of bias will be evaluated using the Cochrane risk of bias 2.0 tool (RoB 2.0) in randomised controlled trials [73], the Cochrane tool for risk of bias in non-randomised studies of interventions (ROBINS-I) in non-randomised studies [74] and the Newcastle-Ottawa Scale (NOS) to assess the quality of cohort and case-control studies [75]. Two reviewers will complete assessments independently for our primary and secondary outcomes of interest across studies. Disagreements will be resolved by discussion and the assessment of a third reviewer, if consensus is not reached. The results from these quality assessments will be detailed in the summary of findings table.

### Data synthesis

A narrative synthesis method will first be used to describe the results of the systematic review. All eligible trials will be summarised in narrative form, and summary of findings tables will be organised according to (a) type of intervention used (i.e. rTMS or tDCS) and (b) disorder. These tables will include key study characteristics (study design, population and intervention parameters, disorder-specific symptoms, mood and neurocognition outcomes). Population and stimulation parameter details for ongoing treatment trials will be detailed in a separate table according to (a) type of intervention being used (i.e. rTMS or tDCS) and (b) disorder.

Then, where possible, meta-analysis methods will be applied. We will use Revman 5.3 software to synthesise and analyse all outcome data. We will use tau-squared and the *I*^2^ test to quantify the statistical heterogeneity between studies examining our outcomes of interest, with *I*^*2*^ values of 25%, 50% and 75% representing low, medium and high heterogeneity, respectively [76]. If feasible and appropriate, outcome data will be used to perform random effects meta-analyses because of heterogeneity is expected a priori. The random effects model assumes the study level effect estimates follow a normal distribution, considering both within-study and between-study variation.

### Subgroup analyses

We will carry out subgroup analyses to test the sources of heterogeneity based on disorder type, intervention type (rTMS or tDCS), study design (e.g., randomised controlled trial or non-randomised controlled trial), intervention duration (number of sessions), illness duration and concomitant treatment (that is, medication, psychological treatment, behavioural treatment or cognitive training).

### Sensitivity analyses

Potential reasons for heterogeneity will be explored in sensitivity analyses; the pre-specified subgroup analyses, if feasible, will be examined to determine potential reasons for any observed statistical heterogeneity.

### Meta-bias and strength of evidence

In order to assess for publication bias, we will use our clinical trials registry search to identify trial protocols which have not published their results and compare published results to their protocols where available. The overall quality of evidence for all outcomes will be evaluated using the Grading, Recommendations, Assessment, Development and Evaluation (GRADE) framework [77], estimating individual risk of bias, meta-bias, precision, consistency, directedness and the magnitude of effect. These indicators will determine the certainty of the estimated effect, which will be rated as either very low, low, high or very high.

## Discussion

In this review, we aim to synthesise the findings of studies addressing the effects of rTMS and tDCS on clinical outcomes in children and young people with psychiatric disorders. It will be based on eligible published studies from inception to present and will allow us to assess study quality and analyse outcome data. It will also provide information on ongoing trials and relevant unpublished studies. Anticipated limitations include the paucity of high-quality trials and insufficient homogeneity of data to perform quantitative analysis. The findings may have valuable implications for multiple stakeholders including patients, health care professionals, health system decision-makers and researchers working in non-invasive brain stimulation. The use of our expected findings by healthcare professionals could contribute to making informed decisions about the choice of therapy. For the research implications, our expected findings could generate relevant research questions related to using NIBS in children and young people with psychiatric disorders.

We plan to disseminate our findings to different audiences including young people and parents of young people with psychiatric disorders, healthcare professionals, researchers and health system decision-makers working in Children and Adolescent Mental Health Services. As we are a clinical academic research group, we have close contact with patient advocacy groups who will be engaged in every step of the dissemination process. The dissemination of our work will consist of publishing our review papers in a peer-reviewed journal, presenting at national and international conferences in the domain of non-invasive brain stimulation and youth mental health and circulating our findings (in plain English) on media networks (e.g. LinkedIn and Twitter).

## Supplementary Information

**Additional file 1.** PRISMA-P 2015 Checklist.

**Additional file 2.** Search strategy draft for Medline (via OVID platform).

**Additional file 3.** Extraction data form.

## Data Availability

Not applicable.

## Abbreviations

rTMS: Repetitive transcranial magnetic stimulation
tDCS: Transcranial direct current stimulation
NIBS: Non-invasive brain stimulation
PRISMA: Preferred reporting items for systematic review and meta-analysis
WHO: World Health Organisation
TMS: Transcranial magnetic stimulation
LTP: Long-term potentiation
LTD: Long-term depression
TBS: Intermittent theta burst stimulation
MDD: Major depressive disorder
ADHD: Attention deficit hyperactivity disorder
ASD: Autism spectrum disorder
LF-rTMS: Low-frequency repetitive transcranial magnetic stimulation
HF-rTMS: High-frequency repetitive transcranial magnetic stimulation
iTBS: Intermittent theta burst stimulation
cTBS: Continuous theta burst stimulation
PAS: Paired associative stimulation
PPS: Paired-pulse stimulation
QPS: Quadripulse stimulation
PANNS: Positive and Negative Syndrome Scale
HDRS: Hamilton Depression Rating Scale
IGT: Iowa Gambling Test
ICTRP: International Clinical Trials Registry Platform
NIH: National Institute of Health
ISRCTN: International Standard Randomised Controlled Trials Number
PRESS: Peer Review of Electronic Search Strategies
RoB 2.0: Risk of Bias 2.0
ROBINS-I: Risk of Bias in Non-randomised Studies of Interventions
NOS: Newcastle-Ottawa Scale
GRADE: Grading, Recommendations, Assessment, Development and Evaluation
