# Supplementary material for: Effects of non-invasive brain stimulation in children and young people with psychiatric disorders: a protocol for a systematic review
Source: Syst Rev. 2021 Mar 11;10:76. doi: 10.1186/s13643-021-01627-3 (PMC7953615; doi:10.1186/s13643-021-01627-3)
Supplement: Supplementary file 2 — Additional file 2. Search strategy draft for Medline (via OVID platform). [file 13643_2021_1627_MOESM2_ESM.docx]

**Additional File 2: Search strategy draft for Medline (via OVID platform)**

| # | **Searches** |
| --- | --- |
| 1 | exp transcranial magnetic stimulation/ |
| 2 | (transcranial magnetic stimulation or TMS or repetitive transcranial magnetic stimulation or rTMS or theta burst stimulation or iTBS or cTBS).tw. |
| 3 | exp transcranial direct current stimulation/ |
| 4 | (transcranial direct current stimulation or tDCS).tw. |
| 5 | 1 or 2 or 3 or 4 |
| 6 | exp child/ |
| 7 | exp adolescent/ |
| 8 | exp young adult/ |
| 9 | (child* or adolescen* or young adult* or youth or boy* or girl* or pediatric* or paediatric or young people or young person*).tw. |
| 10 | 6 or 7 or 8 or 9 |
| 11 | exp autism/ |
| 12 | autis*.tw. |
| 13 | exp attention deficit disorder/ |
| 14 | (attention deficit hyperactivity disorder* or ADHD).tw. |
| 15 | exp conduct disorder/ |
| 16 | exp oppositional defiant disorder/ |
| 17 | exp intermittent explosive disorder/ |
| 18 | (oppositional defiant disorder* or explosive disorder* or conduct disorder* or pyromania or kleptomania or impulse control disorder*).tw. |
| 19 | exp schizophrenia/ |
| 20 | (schizophrenia or psychotic disorder* or schizoaffective disorder* or catatonia).tw. |
| 21 | exp mood disorder/ |
| 22 | exp bipolar disorder/ |
| 23 | exp depression/ |
| 24 | (mood disorder* or affective disorder* or cylothymi* or depress* or bipolar* or dysthymi*).tw. |
| 25 | exp anxiety disorder/ |
| 26 | exp panic/ |
| 27 | (anxiety disorder* or neurotic disorder* or panic disorder* or agoraphobia* or selective mutism or social phobia).tw. |
| 28 | exp obsessive compulsive disorder/ |
| 29 | exp Gilles de la Tourette syndrome/ |
| 30 | (obsessive-compulsive disorder* or OCD or tic disorder* or tourette or hoarding or body dysmorphic disorder* or trichotillomania or excoriation disorder*).tw |
| 31 | exp posttraumatic stress disorder/ |
| 32 | exp acute stress disorder/ |
| 33 | (post-traumatic stress disorder* or post traumatic stress disorder* or PTSD or acute stress disorder*).tw. |
| 34 | exp substance abuse/ |
| 35 | exp substance-related disorders/ |
| 36 | ("substance use" or "substance abuse" or addiction).tw. |
| 37 | exp somatoform disorder/ |
| 38 | (somatoform or somatoform disorder* or somati#ation or conversion disorder* or hypochondri*).tw. |
| 39 | exp eating disorder/ |
| 40 | exp feeding disorder/ |
| 41 | (((eating disorder* or anorexia* or bulimi* or binge-eating* or binge eating* or avoidant restrictive food intake disorder eating disorder* or ARFID or eating disorder) not otherwise specified) or EDNOS or other specified feeding or eating disorder or OSFED).tw. |
| 42 | exp personality disorder/ |
| 43 | (personality disorder* or anti social personality disorder* or anti-social personality disorder* or borderline personality disorder* or emotionally unstable personality disorder* or obsessive-compulsive personality disorder* or obsessive compulsive personality disorder* or dependent personality disorder* or histrionic personality disorder* or narcissistic personality disorder* or avoidant personality disorder* or schizoid personality disorder* or schizotypal personality disorder*).tw. |
| 44 | 11 or 12 or 13 or 14 or 15 or 16 or 17 or 18 or 19 or 20 or 21 or 22 or 23 or 24 or 25 or 26 or 27 or 28 or 29 or 30 or 31 or 32 or 33 or 34 or 35 or 36 or 37 or 38 or 39 or 40 or 41 or 42 or 43 |
| 45 | 5 and 10 and 44 |
