# Supplementary material for: Effects of non-invasive brain stimulation in children and young people with psychiatric disorders: a protocol for a systematic review
Source: Syst Rev. 2021 Mar 11;10:76. doi: 10.1186/s13643-021-01627-3 (PMC7953615; doi:10.1186/s13643-021-01627-3)
Supplement: Supplementary file 3 — Additional file 3. Extraction data form. [file 13643_2021_1627_MOESM3_ESM.docx]

**Additional file 3: Extraction data form**

| **General** | |
| --- | --- |
| Paper title |  |
| Date form completed |  |
| Reference citation |  |
| Publication type |  |
| Is it in English? |  |
| Notes |  |
| **Participants** | |
| Number started |  |
| Number completed |  |
| A priori power calculation |  |
| Age (Mean, SD) and range |  |
| Gender |  |
| Ethnicity |  |
| Inclusion Criteria |  |
| Exclusion Criteria |  |
| Main disorder being treated |  |
| Treatment setting |  |
| Severity of illness |  |
| Group sub-division |  |
| **Intervention – general** | |
| Was neurostimulation given as an add-on treatment? Details of basic treatment |  |
| Was neurostimulation coupled with cognitive or other task? |  |
| **Intervention – TMS** | |
| Type of rTMS used (high-frequency, low-frequency, deep TMS, cTBS, iTBS) |  |
| Number and frequency of sessions |  |
| Site of stimulation |  |
| rTMS frequency (Hz) |  |
| Coil type |  |
| % of resting motor threshold |  |
| Number of trains |  |
| Train duration |  |
| Inter-train interval |  |
| Neuronavigation use? Yes/No |  |
| Compliance with rTMS regimen (number of sessions missed for exclusion) |  |
|  |  |
| **Intervention – tDCS** | |
| tDCS machine used |  |
| Number and frequency of sessions |  |
| Site of stimulation |  |
| Current |  |
| Duration of current |  |
| Compliance with rTMS regimen (number of sessions missed for exclusion) |  |
| **Study details** | |
| Aims |  |
| Study dates |  |
| Study design (case report, open trial, controlled trial etc.) |  |
| Comparison |  |
| Recruitment procures |  |
| Baseline imbalances |  |
| Primary outcome measures |  |
| Secondary outcome measure |  |
| Main clinical assessment tools |  |
| Mood assessment tools |  |
| Cognitive assessment tools |  |
| Assessment time point |  |
| Missing data |  |
| Limitations |  |
| **Results** | |
| Main clinical outcomes |  |
| Secondary outcomes (mood, cognitive) |  |
| Adverse outcomes |  |
| Main conclusion |  |
